# Prediction of prognosis in patients with left ventricular dysfunction using three-dimensional strain echocardiography and cardiac magnetic resonance imaging

**DOI:** 10.1007/s12471-022-01688-6

**Published:** 2022-05-10

**Authors:** M. F. A. Aly, S. A. Kleijn, J. H. van Lenthe, R. F. Menken-Negroiu, L. F. Robbers, A. M. Beek, O. Kamp

**Affiliations:** 1 Department of Cardiology, VU University Medical Centre, Amsterdam UMC, Amsterdam, The Netherlands; 2 Department of Cardiology, University Hospital, Beni-Suef, Egypt

**Keywords:** Speckle tracking echocardiography, Cardiac magnetic resonance, Prognosis, Ventricular function

## Abstract

**Background:**

We evaluated three-dimensional speckle tracking echocardiography (3DSTE) strain and cardiac magnetic resonance (CMR) with delayed contrast enhancement (DCE) for the prediction of cardiac events in left ventricular (LV) dysfunction.

**Methods:**

CMR and 3DSTE in 75 patients with ischaemic and 38 with non-ischaemic LV dysfunction were analysed and temporally correlated to cardiac events during 41 ± 9 months of follow-up.

**Results:**

Cardiac events occurred in 44 patients, more in patients with ischaemic LV dysfunction. LV ejection fraction (LVEF), global circumferential and global area strain were reduced more in patients with more cardiac events, whereas 3DSTE LV end-systolic volumes and 3DSTE LV masses were larger. However, the area under the curve using receiver-operating characteristic analysis showed modest sensitivity and specificity for all evaluated parameters. Additionally, DCE did not differ significantly between the two groups. Univariate analysis showed ischaemic aetiology of LV dysfunction, LVEF and LV mass by CMR to be predictors of cardiac events with an increased relative risk of 2.4, 1.6 and 1.5, respectively. By multivariate analysis, only myocardial ischaemia and LVEF ≤ 39% were independent predictors of events (*p* = 0.004 and 0.005, respectively). Subgroup analysis in ischaemic and non-ischaemic patients showed only 3DSTE LV mass in ischaemic patients to have a significant association (*p* = 0.033) but without an increased relative risk.

**Conclusion:**

LVEF calculated by 3DSTE or CMR were both good predictors of cardiac events in patients with LV dysfunction. A reduced LVEF ≤ 39% was associated with a 1.6-fold higher probability of a cardiac event. 3DSTE strain measurements and DCE-CMR did not add to the prognostic value of LVEF.

**Supplementary Information:**

The online version of this article (10.1007/s12471-022-01688-6) contains supplementary material, which is available to authorized users.

## What’s new?

- We present a novel study to test the ability of three-dimensional speckle tracking echocardiography to predict adverse cardiac events in patients with left ventricular (LV) dysfunction.
- Patients with ischaemic and non-ischaemic LV dysfunction were studied and compared.
- Cardiac magnetic resonance (CMR) with delayed contrast enhancement (DCE), a well-studied imaging modality, was used for this purpose.
- A relatively new objective method of quantifying DCE-CMR was applied according to the most recent recommendation.

## Introduction

Left ventricular (LV) systolic dysfunction is a major cause of morbidity and mortality. LV ejection fraction (LVEF) is a well-established prognostic parameter of cardiac outcomes [1]. However; the currently used two-dimensional measurements have limitations related to geometric assumptions, reproducibility and expertise [2]. Cardiac magnetic resonance (CMR) is the reference technique for LV chamber quantification and tissue characterisation through delayed contrast enhancement (DCE), which evaluates the structural extent of fibrosis and has been shown to be a good predictor of adverse prognosis in different myocardial diseases [3]. The accuracy of three-dimensional speckle tracking echocardiography (3DSTE) for LV chamber quantification has been clinically validated against CMR [4, 5], and a direct comparison of the two techniques for identification of myocardial transmural scar has recently been presented [5]. Novel strain parameters using 3DSTE are able to quantify the complex myocardial mechanics and may predict LV remodelling [6, 7]. In the current study, we evaluated the prognostic value of both imaging techniques in patients with LV systolic dysfunction.

## Methods

### Study design and population

This is an observational study involving 113 patients, referred to the VU University Medical Centre (VUmc) between February 2010 and December 2011 for CMR using DCE, and three-dimensional echocardiography was performed on the same day. The patients were followed up to register the occurrence of subsequent cardiac events until November 2014 using their medical records from VUmc and from another 15 hospitals all over the Netherlands. All patients received optimal medical treatment in addition to coronary revascularisation and device implantation, if needed, according to practice guidelines. Informed consent was obtained from patients or their relatives in the case of death and from their hospitals.

Initially, 148 consecutive patients with sinus rhythm and LV systolic dysfunction (EF < 50% by two-dimensional echocardiography) were included. Ischaemic patients were defined by coronary angiography showing > 50% reduction of vessel diameter in one or more major coronary arteries or a history of myocardial infarction and/or coronary revascularisation. Twenty-eight patients were excluded due to poor image quality (25 with 3DSTE and 3 with CMR) and 7 patients were lost to follow-up. Of the remaining 113 patients; 75 patients had ischaemic and 38 had idiopathic non-ischaemic LV dysfunction.

### CMR and echocardiographic analysis

**Fig. 1:**
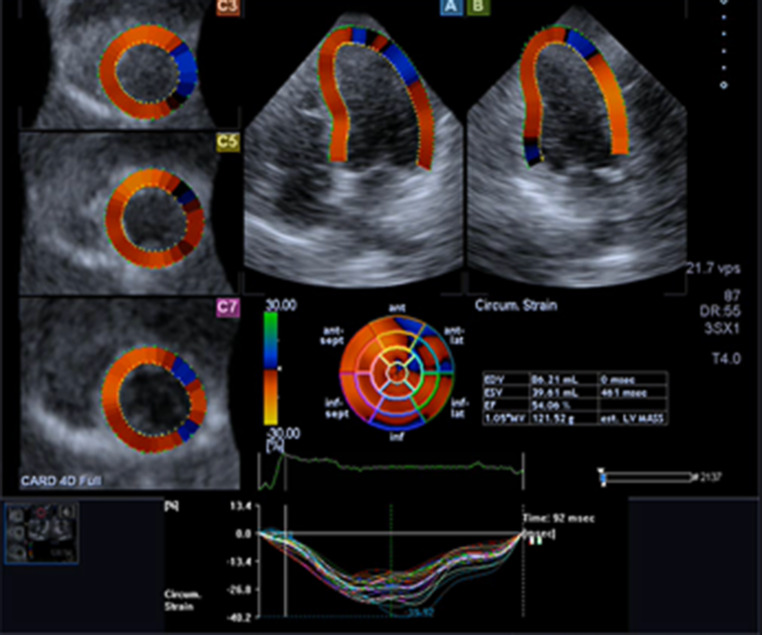
Measurements of the left ventricular (*LV*) volumes and function using three-dimensional speckle tracking echocardiography. Example of multiple long- and short-axis views extracted from a three-dimensional echocardiography data set at end-systole in a patient with normal systolic LV function. Visual information on circumferential (*Circum.*) strain is shown in colour overlay superimposed on grey-scale images (*EDV* end-diastolic volume, *ESV* end-systolic volume, *EF* ejection fraction)

Imaging and analysis of CMR and 3DSTE were performed as previously described [4, 5]. LV 3DSTE strain data included conventional strain components (global circumferential strain (GCS), global longitudinal (GLS) and global radial strain) and global area strain (GAS) ([5, 6]; Fig. 1). All echocardiographic and DCE-CMR analyses were performed separately and blinded to other analyses and patients’ data. (Electronic Supplementary Material, Table S1 compares 3DSTE and CMR techniques).

### Cardiac events

Cardiac events were recorded from patients’ files and defined as the occurrence of all-cause mortality, myocardial infarction, and revascularisation, hospitalisation for heart failure or device implantation. Myocardial infarction included both ST-elevation myocardial infarction (STEMI) and non-STEMI. Revascularisation included elective and urgent percutaneous coronary intervention and coronary artery bypass graft. Device implantation included the placement of a biventricular pacemaker for cardiac resynchronisation therapy and/or an implantable cardioverter-defibrillator for primary or secondary prevention according to current guidelines.

### Statistical analysis

Continuous variables are presented as mean ± SD, whereas categorical variables are presented as frequencies and percentages. We used Student’s *t*-test to compare means among groups, the chi-square test or Fisher’s exact test to analyse the distribution of categorical variables and Spearman’s test to correlate numerical variables. Variables that were statistically significant on univariate analysis with a *p-*value < 0.05 were entered in a forward conditional stepwise multivariate logistic regression model to detect independent predictors of cardiac events for all patients as well as for ischaemic and non-ischaemic subgroups. Receiver-operating characteristic (ROC) curve analysis was done to find the optimal cut-off values of statistically significant continuous variables using the Youden index, with calculation of sensitivity, specificity and relative risk. Cardiac event-free survival rates were calculated on the basis of a Kaplan-Meier analysis and were compared among patient groups with the log rank test. All tests were bilateral and a *p*-value < 0.05 was considered statistically significant. All data were analysed using SPSS version 24.0 for Mac (IBM Inc., Chicago, IL, USA).

## Results

### Patients’ characteristics

Most patients were male (72%) and two-thirds (67%) had an ischaemic LV dysfunction. The mean age of the patients was 61 years and the mean LVEF was 39%. DCE was positive in 75 (66%) patients. Patients with cardiac events had more ischaemic LV dysfunction (*n* = 35; 80%). No significant difference was present between patients with and those without cardiac events regarding age, gender, the presence of hypertension or diabetes (Table 1).

**Table 1:** Risk factors associated with cardiac events in all patients

Variable	Cardiac events		*p‑value*
	Yes (*n* = 44, 39%)	No (*n* = 69, 61%)	
*Patients’ characteristics*			
Age (years)	64 ± 11	61 ± 14	0.115
Male (*n*, %)	33, 75	48, 70	0.532
HR (bpm)	67 ± 6	66 ± 5	0.785
BSA (kg/m^2^)	1.9 ± 0.2	1.9 ± 0.2	0.789
Hypertension (*n*, %)	22, 50	26, 38	0.196
Diabetes mellitus (*n*, %)	7, 16	12, 17	0.837
Patients with CRT/ICD/CRT‑D	22, 29	12, 32	0.806
Duration of follow-up	42 ± 9	41 ± 8	0.737
Ischaemic LV dysfunction (*n*, %)	35, 80	40, 58	0.018*
*CMR*			
LVEDV (ml)	218 ± 61	207 ± 65	0.345
LVESV (ml)	145 ± 64	126 ± 62	0.136
LVEF (%)	36 ± 12	41 ± 13	0.023*
LV mass (g)	114 ± 30	114 ± 37	0.972
*DCE*			
Positive (*n*, %)	31, 41	44, 59	0.463
Enhanced mass (g)	114 ± 30	114 ± 37	0.390
% Enhanced mass	12 ± 12	10 ± 13	0.390
*3DSTE*			
LVEDV (ml)	164 ± 60	145 ± 49	0.086
LVESV (ml)	113 ± 61	89 ± 46	0.019*
LVEF (%)	34 ± 13	41 ± 13	0.008*
LV mass (g)	169 ± 42	151 ± 35	0.018*
GLS (%)	−9 ± 4	−10 ± 4	0.129
GCS (%)	−15 ± 6	−18 ± 7	0.034*
GRS (%)	18 ± 10	20 ± 9	0.219
GAS (%)	−22 ± 8	−26 ± 9	0.017*

*3DSTE* three-dimensional speckle tracking echocardiography, *BSA* body surface area, *CRT‑D* cardiac resynchronisation therapy-defibrillator, *CMR* cardiac magnetic resonance, *DCE* delayed contrast enhancement, *ICD* implanted cardioverter-defibrillator, *GAS* global area strain, *GCS* global circumferential strain, *GLS* global longitudinal strain, *GRS* global radial strain, *HR* heart rate, *LV* left ventricular, *LVEDV* left ventricular end-diastolic volume, *LVESV* left ventricular end-systolic volume, *LVEF* left ventricular ejection fraction

*Difference is significant at the 0.05 level

### 3DSTE and CMR risk parameters for cardiac events

Patients having cardiac events had significantly larger 3DSTE-derived LV end-systolic volume and LV mass, lower LVEF, lower GCS and GAS compared to patients without cardiac events. For CMR, there was a lower LVEF in patients with cardiac events while other CMR parameters, including percentage or total amount of DCE, failed to show a significant difference between the two patient groups. Subgroup analysis in patients with ischaemic and non-ischaemic LV dysfunction revealed that LVEF, 3DSTE LV end-systolic volume and LV mass showed a significant difference between patients with and those without cardiac events in the ischaemic group, whereas in the non-ischaemic group only LVEF was significantly different (Table 2).

**Table 2:** Risk factors associated with cardiac events: subgroup analysis of ischaemic and non-ischaemic patients

(I) Ischaemic patients (*n* = 74)	Patients with cardiac events (*n* = 35, 47%)	Patients without cardiac events (*n* = 39, 53%)	*p‑value*
*CMR*			
LVEF (%)	33 ± 12	43 ± 12	0.041*
*3D STE*			
LVEF (%)	36 ± 13	42 ± 12	0.027*
LVESV (ml)	108 ± 63	82 ± 41	0.036*
LV mass (g)	169 ± 43	149 ± 35	0.031*
GCS (%)	−16 ± 6	−18 ± 7	0.120
GAS (%)	−23 ± 8	−27 ± 9	0.520
(II) Non-ischaemic patients (*n* = 38)	Patients with cardiac events (*n* = 8, 21%)	Patients without cardiac events (*n* = 30, 79%)	*p‑value*
*CMR*			
LVEF (%)	29 ± 13	40 ± 14	0.048*
*3D STE*			
LVEF (%)	29 ± 14	40 ± 14	0.049*
LVESV (ml)	135 ± 53	98 ± 51	0.074
LV mass (g)	171 ± 40	155 ± 36	0.270
GCS (%)	−11 ± 6.2	−17 ± 8	0.066
GAS (%)	−19 ± 9	−26 ± 10	0.078

*3DSTE* three-dimensional speckle tracking echocardiography, *CMR* cardiac magnetic resonance, *GAS* global area strain, *GCS* global circumferential strain, *GRS* global radial strain, *LV* left ventricular, *LVESV* left ventricular end-systolic volume, *LVEF* left ventricular ejection fraction

*Difference is significant at the 0.05 level

### Cardiac events

Over a follow-up period of 41 ± 9 months, a total of 61 cardiac events occurred in 44 patients and multiple cardiac events occurred in 11 patients (Table 3).

**Table 3:** Cardiac events in all patients during follow-up

Cardiac event	*n*
*Mortality*	9
Cardiac cause	3
Non-cardiac cause	3
Unknown cause	3
*Hospitalisation for heart failure*	9
*Myocardial infarction*	12
STEMI	3
NSTEMI	9
*Revascularisation*	20
Urgent PCI	4
Elective PCI	7
Elective CABG	9
*New device implantation*	11
CRT/CRT‑D	5
ICD	6

*CABG* coronary artery bypass graft, *CRT‑D* cardiac resynchronisation therapy-defibrillator, *ICD* implanted cardioverter-defibrillator, *NSTEMI* non-ST-elevation myocardial infarction, *STEMI* ST-elevation myocardial infarction, *PCI* percutaneous coronary intervention

### Predictive value of 3DSTE and CMR

Three parameters, namely ischaemic aetiology of the LV dysfunction (*p* = 0.008), LVEF measured by either 3DSTE (*p* = 0.02) or CMR (*p* = 0.04), and LV mass (*p* = 0.04) were significant predictors with univariate regression analysis. By applying a stepwise multivariate logistic regression analysis, only the presence of an ischaemic aetiology and LVEF ≤ 39% were independent predictors of cardiac events in all patients (*p* = 0.004, 0.005 and 0.007, respectively). When repeating this analysis separately in the ischaemic and non-ischaemic patient subgroups, LV mass > 150 g was the only independent predictor of cardiac events in ischaemic patients only (*p* = 0.033) (Electronic Supplementary Material, Table S2).

**Fig. 2:**
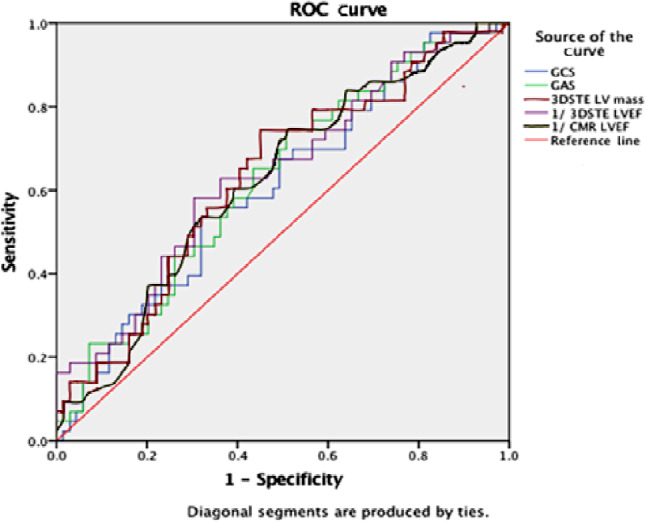
Receiver-operating characteristic (*ROC*) curve analysis for the prediction of cardiac events. Left ventricular ejection fraction (*LVEF*) is the most sensitive parameter with a sensitivity of 61% (*3DSTE* three-dimensional speckle tracking echocardiography, *CMR* cardiac magnetic resonance, *GAS* global area strain, *GCS* global circumferential strain)

**Fig. 3:**
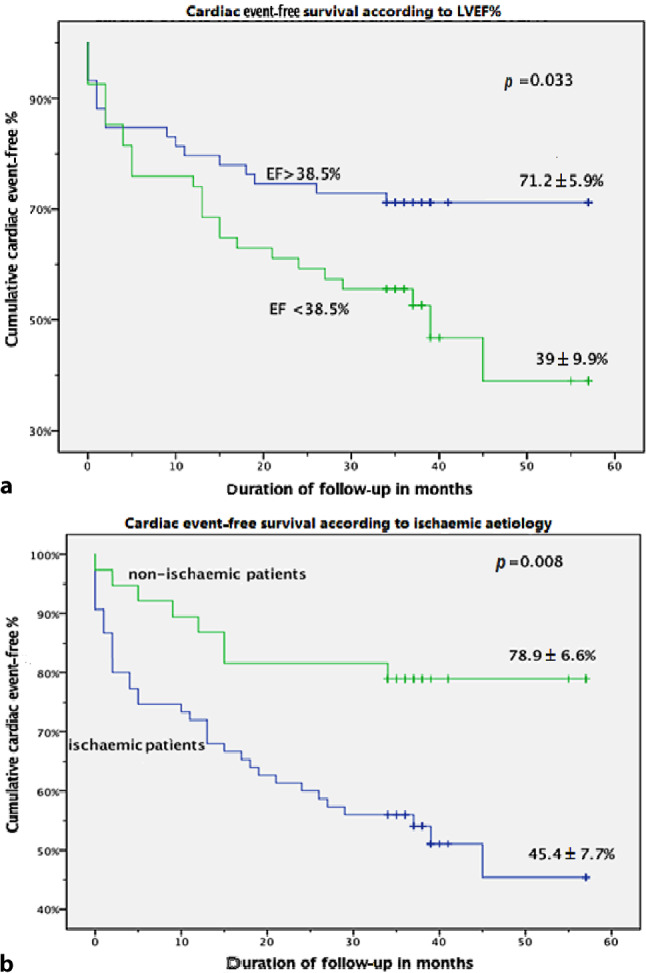
Kaplan-Meier curves showing freedom from cardiac events regarding different parameters: **a** according to left ventricular ejection fraction percentage (*LVEF%*), **b** according to ischaemic aetiology. Data are dichotomised on the basis of the calculated cut-off value of each parameter

Using the ROC curve analysis to define the best sensitivity and specificity of these significant predictors, the area under the curve was below 0.7 for all parameters. For LVEF, the area under the curve was only 0.64 ± 0.05 and 0.62 ± 0.05 calculated by either 3DSTE or CMR with *p* = 0.013 and *p* = 0.032, respectively. The sensitivity and specificity of a low LVEF of ≤ 39% was 61–63% and 61%; respectively (Fig. 2). As demonstrated in the Kaplan Meier curve, the cumulative probability of a cardiac event-free survival at 5 years for ischaemic patients was significantly smaller than that for non-ischaemic patients (*p* = 0.008). At 5 years, the cumulative probability of a cardiac event-free survival calculated for patients with an LVEF of ≤ 39% was significantly lower than that of patients with an LVEF > 39% (39 ± 10% vs 71 ± 6%, respectively; *p* = 0.033) (Fig. 3).

There were good correlations between strain and other LV structural and functional parameters with the best correlation between LVEF and GAS (*r* = 0.98). Reduced strain was associated with larger LV volumes and lower LVEF. As expected, the measurements of LVEF by 3DSTE and CMR correlated strongly (*r* = 0.87).

## Discussion

3DSTE represents an advancement in echocardiography towards more comprehensive LV chamber quantification, including volumes and LVEF with more advanced parameters assessing myocardial deformation with 3D strain. The aim of this study was to test the ability of 3DSTE strain to predict subsequent cardiac events in patients with impaired LVEF compared to CMR using DCE. Our results showed that prognostic information is similar between 3DSTE- and CMR-derived LVEF and 3DSTE strains, and DCE-CMR did not add to the prognostic power of LVEF.

### Predictive value of global 3D strain

Previous studies using two-dimensional speckle tracking echocardiography (2DSTE) revealed that the sub-endocardial fibres, responsible the long axis contraction, are often affected first regardless of the pathophysiology, leading to decreased GLS before clinical symptoms and reduction in LVEF occur [3, 8, 9]. With progression of the disease, the mid-myocardial fibres, which are oriented mainly circumferentially with a major contribution to circumferential LV function and LV myocardial thickening greater than that of longitudinal fibres, are affected as well, leading to impairment of the circumferential and then the radial strain [10] (Electronic Supplementary Material, Fig. S1). Therefore, the longitudinal strain could be a sensitive marker of early myocardial dysfunction, whereas impaired circumferential and radial strain, which denote more advanced transmural interference and more myocardial dysfunction, may be more closely related to prognosis [3]. Some 2DSTE studies reported that GCS was independently associated with all-cause mortality [11] and a better parameter than LVEF for prognostic stratification in patients hospitalised for acute heart failure [10] as well as a better parameter than LVEF and DCE-CMR in patients with ischaemic cardiomyopathy [12]. Moreover, Matsumoto et al. found 3D GCS to be a good predictor in patients with non-ischaemic cardiomyopathy [13]. Their results could be explained by the finding that approximately 30% of those patients have mid-wall fibrosis, which impairs the circumferential shortening [6]. GAS, which mainly reflects the myocardial changes in the mid-layer, was demonstrated to be a reliable and reproducible surrogate of LVEF [6] and has been shown to be able to predict and quantify the transmural extent of scar [14]. Moreover, GAS might have the potential to detect myocardial viability in patients with ischaemic LV dysfunction [15]. Our analysis revealed that 3D GCS and GAS showed the best correlation (*r* = −0.96 and −0.98, respectively) with LVEF, which is comparable to the results from a previous study [6]. Moreover, both GCS and GAS were the only 3D strains that were significantly lower in patients who developed cardiac events compared to those who did not. However, contrary to previous results, none of these strain parameters were able to add to the prognostic power of LVEF. Although several studies have shown GLS either by 2DSTE [3, 8, 9] or 3DSTE [16] to offer a high predictive value in a variety of myocardial conditions such as ischaemic and non-ischaemic LV dysfunction, our results did not support these findings as GLS was similarly impaired in both patient groups. This result is in line with previous studies which yielded no prognostic significance of GLS derived by 2DSTE [10] or 3DSTE [17]. Of note is that some studies showed a weak correlation between GLS by both techniques and this could be related to their different inherit characteristics [18].

LVEF is the most important clinical parameter to assess LV systolic function, on which many clinical decisions depend, such as when to initiate medical therapy, to guide cardiac devices or to perform cardiac interventions. LVEF is currently the most frequently used parameter to predict cardiac events [19, 20]. Ischaemic cardiomyopathy is the most common cause of heart failure in developed countries and has a poor outcome, worse than non-ischaemic forms of dilated cardiomyopathy. This is related to the higher risk of ventricular arrhythmias, recurrent ischaemia, new myocardial infarctions and systemic complications [21]. Increased LV end-systolic volume has been considered a risk marker for developing heart failure and used for selection of surgical intervention in mitral and aortic regurgitation [22], and its decrease is a measure of response to cardiac resynchronisation therapy [23]. Increased LV mass as a surrogate of cardiac remodelling and increased afterload has been proved to be a powerful and independent predictor of increased morbidity and mortality in heart diseases [20]. The present study confirmed the importance of these parameters as established predictors of cardiac events, particularly LVEF.

### Predictive value of 3DSTE versus DCE-CMR

CMR and DCE are currently the reference techniques for LV chamber quantification and the identification of the site and extent of myocardial fibrosis. DCE indicates irreversible myocardial damage, including myocardial infarction [24], or focal myocardial fibrosis from non-ischaemic causes [25]. The presence of DCE was associated with unfavourable prognosis regardless of the aetiology of fibrosis [2, 3]. 2DSTE GLS was markedly attenuated regardless of the extent of myocardial fibrosis, as delineated by DCE-CMR [5, 26]. The correlation between global or regional STE strain and DCE-CMR was generally poor [5, 13, 26]. When 3DSTE strain was correlated to myocardial infarct size by CMR, all Pearson’s correlation coefficients were < 0.5, which should be interpreted as moderate to poor [5, 14]. In the present study, some 3DSTE strains are functional parameters that are better able than DCE-CMR to differentiate between patients with cardiac events and those without. A possible explanation for this negative result for DCE could be that all of the previous studies used a threshold of 2 SD above the mean signal intensity of normal myocardium and they assessed the presence and amount of DCE visually, which is subjective and moderately reproducible. In the present study we used a dedicated software for objective quantification of DCE using the recently recommended higher threshold of 5 SD. It was shown to best correlate to the visual analysis of DCE images and has similar ability to other thresholding techniques in predicting segmental functional improvement after revascularisation [27–29].

### Study limitations

The main limitations of this study are the relatively small cohort size and the medium duration of follow-up. Due to the retrospective design with follow-up performed through patients’ files, certain baseline characteristics could not be identified and there may have been an under-reporting of cardiac events. In addition, the current lack of standardisation among different ultrasound machines and software provided by different vendors may preclude generalisation of our results [30]. Nonetheless, this is a novel study comparing 3DSTE with DCE for predicting subsequent cardiac events in patients with ischaemic and non-ischaemic LV dysfunction. A larger prospective study with similar clinical outcomes is warranted.

## Conclusions

LVEF calculated using 3DSTE was a good predictor of subsequent cardiac events in patients with LV dysfunction. A reduced LVEF < 39% was associated with a 1.6-fold higher probability of a cardiac event. However, 3DSTE strains and DCE-CMR did not add to the prognostic power of LVEF.

## Supplementary Information

**Table S1 **Comparison between 3DSTE and CMR techniques

**Table S2 **Results of univariate and multivariate logistic regression analysis for predictors of cardiac events

**Fig. S1 Cardiac magnetic resonance delayed contrast enhancement and three-dimensional speckle tracking echocardiography radial strain in a patient with transmural infraction**. (**a**) Image of a patient with akinesia and transmural infarction of the septal and anterior walls (> 50% hyperenhancement) and (**b**) Colour-coded short-axis 3DSTE radial strain image at end-systole, radial strain is decreased as depicted by a blue colour overlay in a comparable region to the hyperenhancement in the CMR DCE, yet this area seems somewhat larger than the DCE one, involving the inferior wall as well. There is reddish colourisation in the other normal contracting segments with no hyperenhancement [5].

## References

[1] McDermott MM, Feinglass J, Lee PI (1997). Systolic function, readmission rates, and survival among consecutively hospitalized patients with congestive heart failure. Am Heart J.

[2] de Haan S, de Boer K, Commandeur J (2014). Assessment of left ventricular ejection fraction in patients eligible for ICD therapy: discrepancy between cardiac magnetic resonance imaging and 2D echocardiography. Neth Heart J.

[3] Kuruvilla S, Adenaw N, Katwal AB (2014). Late gadolinium enhancement on cardiac magnetic resonance predicts adverse cardiovascular outcomes in non-ischemic cardiomyopathy: a systematic review and meta-analysis. Circ Cardiovasc Imaging.

[4] Kleijn SA, Brouwer WP, Aly MF (2012). Comparison between three-dimensional speckle-tracking echocardiography and cardiac magnetic resonance imaging for quantification of left ventricular volumes and function. Eur Heart J Cardiovasc Imaging.

[5] Aly MFA, Kleijn SA (2016). Three-dimensional speckle tracking echocardiography and cardiac magnetic resonance for left ventricular chamber quantification and identification of myocardial transmural scar. Neth Heart J.

[6] Kleijn SA, Aly MF, Terwee CB (2011). Three-dimensional speckle tracking echocardiography for automatic assessment of global and regional left ventricular function based on area strain. J Am Soc Echocardiogr.

[7] Altman M, Bergerot C, Aussoleil A (2014). Assessment of left ventricular systolic function by deformation imaging derived from speckle tracking: a comparison between 2D and 3D echo modalities. Eur Heart J Cardiovasc Imaging.

[8] Wang J, Khoury DS, Yue Y (2008). Preserved left ventricular twist and circumferential deformation, but depressed longitudinal and radial deformation in patients with diastolic heart failure. Eur Heart J.

[9] Bertini M, Ng AC, Antoni ML (2012). Global longitudinal strain predicts long-term survival in patients with chronic ischemic cardiomyopathy. Circ Cardiovasc Imaging.

[10] Cho GY, Marwick TH, Kim HS (2009). Global 2-dimensional strain as a new prognosticator in patients with heart failure. J Am Coll Cardiol.

[11] Hung CL, Verma A, Uno H (2010). Longitudinal and circumferential strain rate, left ventricular remodelling, and prognosis after myocardial infarction. J Am Coll Cardiol.

[12] Hamada S, Schroeder J, Hoffmann R (2016). Prediction of outcomes in patients with chronic ischemic cardiomyopathy by layer-specific strain echocardiography: a proof of concept. J Am Soc Echocardiogr.

[13] Matsumoto K, Tanaka H, Kaneko A (2012). Contractile reserve assessed by three-dimensional global circumferential strain as a predictor of cardiovascular events in patients with idiopathic dilated cardiomyopathy. J Am Soc Echocardiogr.

[14] Hayat D, Kloeckner M, Nahum J (2012). Comparison of real-time three-dimensional speckle tracking to magnetic resonance imaging in patients with coronary heart disease. Am J Cardiol.

[15] Ran H, Zhang PY, Zhang YX (2016). Assessment of left ventricular myocardial viability by 3-dimensional speckle-tracking echocardiography in patients with myocardial infarction. J Ultrasound Med.

[16] Nagata Y, Takeuchi M, Wu VC (2015). Prognostic value of LV deformation parameters using 2D and 3D speckle-tracking echocardiography in asymptomatic patients with severe aortic stenosis and preserved LV ejection fraction. JACC Cardiovasc Imaging.

[17] Kowalik E, Kowalski M, Klisiewicz A (2016). Global area strain is a sensitive marker of subendocardial damage in adults after optimal repair of aortic coarctation: three-dimensional speckle-tracking echocardiography data. Heart Vessels.

[18] Poyraz E, Oz TK, Güvenç RC (2019). Correlation and agreement between 2D and 3D speckle-tracking echocardiography for left ventricular volumetric, strain, and rotational parameters in healthy volunteers and in patients with mild mitral stenosis. Echocardiography.

[19] Solomon SD, Anavekar N, Skali H (2005). Influence of ejection fraction on cardiovascular outcomes in a broad spectrum of heart failure patients. Circulation.

[20] Quinones MA, Greenberg BH, Kopelen HA (2000). Echocardiographic predictors of clinical outcome in patients with left ventricular dysfunction enrolled in the SOLVD registry and trials: significance of left ventricular hypertrophy. Studies of left ventricular dysfunction. J Am Coll Cardiol.

[21] Køber L, Thune JJ, Nielsen JC (2016). Defibrillator implantation in patients with non-ischemic systolic heart failure. N Engl J Med.

[22] Nishimura RA, Otto CM, Bonow RO (2014). 2014 AHA/ACC guideline for the management of patients with valvular heart disease: a report of the American College of Cardiology/American HeartAssociation Task Force on Practice Guidelines. Circulation.

[23] Aly MF, Kleijn SA, de Boer K (2013). Comparison of three-dimensional echocardiographic software packages for assessment of left ventricular mechanical dyssynchrony and prediction of response to cardiac resynchronization therapy. Eur Heart J Cardiovasc Imaging.

[24] Kim RJ, Albert TS, Wible JH (2008). Performance of delayed-enhancement magnetic resonance imaging with gadoversetamide contrast for the detection and assessment of myocardial infarction: an international, multicenter, double-blinded, randomized trial. Circulation.

[25] Klem I, Shah DJ, White RD (2011). Prognostic value of routine cardiac magnetic resonance assessment of left ventricular ejection fraction and myocardial damage: an international, multicenter study. Circ Cardiovasc Imaging.

[26] Kansal MM, Panse PM, Abe H (2012). Relationship of contrast-enhanced magnetic resonance imaging-derived intramural scar distribution and speckle tracking echocardiography-derived left ventricular two-dimensional strains. Eur Heart J Cardiovasc Imaging.

[27] Schulz-Menger J, Bluemke DA, Bremerich J (2013). Standardized image interpretation and post processing in cardiovascular magnetic resonance: Society for Cardiovascular Magnetic Resonance (SCMR) Board of Trustees Task Force on Standardized Post-Processing. J Cardiovasc Magn Reson.

[28] Schulz-Menger J, Bluemke DA, Bremerich J (2020). Standardized image interpretation and post-processing in cardiovascular magnetic resonance—2020 update: Society for Cardiovascular Magnetic Resonance (SCMR): Board of Trustees Task Force on Standardized Post-Processing. J Cardiovasc Magn Reson.

[29] Bojer AS, Sørensen MH, Vejlstrup N (2020). Distinct non-ischemic myocardial late gadolinium enhancement lesions in patients with type 2 diabetes. Cardiovasc Diabetol.

[30] Badano LP, Cucchini U, Muraru D (2013). Use of three-dimensional speckle tracking to assess left ventricular myocardial mechanics: inter-vendor consistency and reproducibility of strain measurements. Eur Heart J Cardiovasc Imaging.

