# Supplementary material for: Prediction of prognosis in patients with left ventricular dysfunction using three-dimensional strain echocardiography and cardiac magnetic resonance imaging
Source: Neth Heart J. 2022 May 10;30(12):572–9. doi: 10.1007/s12471-022-01688-6 (PMC9691806; doi:10.1007/s12471-022-01688-6)
Supplement: Supplementary file 1 — Table S1 Comparison between 3DSTE and CMR techniques [file 12471_2022_1688_MOESM1_ESM.docx]

**Table S1**Comparison between 3DSTE and CMR techniques

| **Variable** | **3DSTE** | **CMR** |
| --- | --- | --- |
| Scanning time | shorter | longer |
| Analysis time | shorter | longer |
| Image quality | moderate | good |
| Spatial resolution | lower | higher |
| Temporal resolution | lower | higher |
| Cost | less | higher |
| LV volumes | accurate with significant underestimation *(cannot differentiate between myocardium and trabeculae)*, well correlated with CMR but cannot be used interchangeably with it | reference technique *(with potential overestimation)* |
| LV EF | -accurate with some underestimation *(low frame rates could miss the actual end-systolic frame)*  -highly comparable with MRI | reference technique |
| LV mass | overestimation (difficult to delineate epicardium) | reference technique |
| LV mechanics (as strain) | -more clinically used  -bigger values  -different reference values however; reflects LV myocardial deformation in a very similar fashion to CMR | -negligible clinical use  -smaller values |
| Identification of myocardial scar | evaluates functional consequences of myocardial fibrosis | evaluates both functional consequences of myocardial fibrosis through strain and anatomical extent through DCE |
| Correlation between 3DSTE strain and CMR DCE for identification of myocardial scar | moderate to poor correlations with CMR DCE | moderate to poor correlations with 3DSE strains |
| Reliability | good | excellent |

3DSTE, three-dimensional speckle tracking echocardiography. BSA, body surface area. CRT-D, cardiac resynchronization therapy-defibrillator. CMR, cardiac magnetic resonance. DCE, delayed contrast enhancement. LV, left ventricular. LVEF, left ventricular ejection fraction. LV mass, left ventricular mass.
