# Supplementary material for: Prediction of prognosis in patients with left ventricular dysfunction using three-dimensional strain echocardiography and cardiac magnetic resonance imaging
Source: Neth Heart J. 2022 May 10;30(12):572–9. doi: 10.1007/s12471-022-01688-6 (PMC9691806; doi:10.1007/s12471-022-01688-6)
Supplement: Supplementary file 2 — Table S2 Results of univariate and multivariate logistic regression analysis for predictors of cardiac events [file 12471_2022_1688_MOESM2_ESM.docx]

**Table S2** Results of univariate and multivariate logistic regression analysis for

predictors of cardiac events

|  | **Univariate analysis** | | **Multivariate analysis** | |
| --- | --- | --- | --- | --- |
| **Variable** | **RR (95% CI)** | ***p*- Value** | **B (SE)** | ***p*- Value** |
| **All patients (n, 113)** | | | | |
| Ischemic LV dysfunction | 2.39 (1.209-4.730) | 0.008* | 1.409(0.491) | 0.004 |
| CMR LVEF†≤ 39% | 1.49 (0.995-2.225) | 0.038* | –0.042(0.010) | 0.007 |
| 3DSTE LVEF†≤ 39% | 1.58 (1.038-2.392) | 0.021* | –0.048(0.017) | 0.005 |
| 3DSTE LV mass†≥155 (g) | 1.49 (0.995-2.225) | 0.038* |  |  |
| **Ischemic patients (n, 75)** | | | | |
| CMR LVEF†≤ 39% | 1.41 (0.963-2.128) | 0.049* |  |  |
| 3DSTE LVEF†≤ 39% | 1.48 (0.933-2.338) | 0.027* |  |  |
| 3DSTE LV mass†≥155 (g) | 1.54 (0.979-2.418) | 0.026* | 0.014(0.007) | 0.033 |
| **Non-ischemic patients (n, 38)** | | | | |
| CMR LVEF†≤ 39% | 4.27 (0.662-27.509) | 0.046* |  |  |
| 3DSTE LVEF†≤ 39% | 2.40 (0.698-8.254) | 0.047* |  |  |
| 3DSTE LV mass†≥155 (g) | 1.51 (0.439-10.830) | 0.335 |  |  |

†, cut-off values as acquired through ROC curve analysis; B, regression coefficient; CI= confidence interval;

RR, relative risk; SE, standard error. Other abbreviations as before.
