# Supplementary material for: Prediction of prognosis in patients with left ventricular dysfunction using three-dimensional strain echocardiography and cardiac magnetic resonance imaging
Source: Neth Heart J. 2022 May 10;30(12):572–9. doi: 10.1007/s12471-022-01688-6 (PMC9691806; doi:10.1007/s12471-022-01688-6)
Supplement: Supplementary file 3 — Fig. S1 Cardiac magnetic resonance delayed contrast enhancement and three-dimensional speckle tracking echocardiography radial strain in a patient with transmural infraction. (a) Image of a patient with akinesia and transmural infarction of the septal and anterior walls (> 50% hyperenhancement) and (b) Colour-coded short-axis 3DSTE radial strain image at end-systole, radial strain is decreased as depicted by a blue colour overlay in a comparable region to the hyperenhancement in the CMR DCE, yet this area seems somewhat larger than the DCE one, involving the inferior wall as well. There is reddish colourisation in the other normal contracting segments with no hyperenhancement [5]. [file 12471_2022_1688_MOESM3_ESM.docx]

**
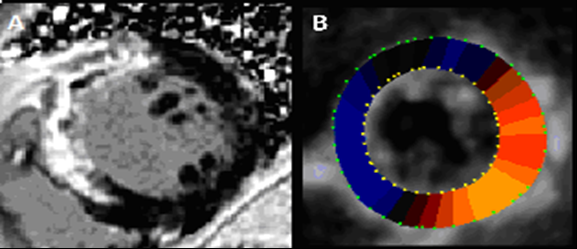
**

**Fig. S1 Cardiac magnetic resonance delayed contrast enhancement and three-dimensional speckle tracking echocardiography radial strain in a patient with transmural infraction**. (**a)** Image of a patient with akinesia and transmural infarction of the septal and anterior walls (>50% hyperenhancement) and (**b**) Colour-coded short-axis 3DSTE radial strain image at end-systole, radial strain is decreased as depicted by a blue colour overlay in a comparable region to the hyperenhancement in the CMR DCE, yet this area seems somewhat larger than the DCE one, involving the inferior wall as well. There is reddish colourisation in the other normal contracting segments with no hyperenhancement [5].
